# Evidence against observational spatial memory for cache locations of conspecifics in marsh tits *Poecile palustris*

**DOI:** 10.1007/s00265-016-2264-2

**Published:** 2017-01-10

**Authors:** A. Utku Urhan, Ellen Emilsson, Anders Brodin

**Affiliations:** Department of Biology, Lund University, Ecology Building, S-223 62 Lund, Sweden

**Keywords:** Observational learning, Spatial memory, Cache pilfering, Scatter hoarding, Food hoarding, Paridae

## Abstract

**Abstract:**

Many species in the family Paridae, such as marsh tits *Poecile palustris*, are large-scale scatter hoarders of food that make cryptic caches and disperse these in large year-round territories. The perhaps most well-known species in the family, the great tit *Parus major*, does not store food itself but is skilled in stealing caches from the other species. We have previously demonstrated that great tits are able to memorise positions of caches they have observed marsh tits make and later return and steal the food. As great tits are explorative in nature and unusually good learners, it is possible that such “memorisation of caches from a distance” is a unique ability of theirs. The other possibility is that this ability is general in the parid family. Here, we tested marsh tits in the same experimental set-up as where we previously have tested great tits. We allowed caged marsh tits to observe a caching conspecific in a specially designed indoor arena. After a retention interval of 1 or 24 h, we allowed the observer to enter the arena and search for the caches. The marsh tits showed no evidence of such observational memorization ability, and we believe that such ability is more useful for a non-hoarding species. Why should a marsh tit that memorises hundreds of their own caches in the field bother with the difficult task of memorising other individuals’ caches? We argue that the close-up memorisation procedure that marsh tits use at their own caches may be a different type of observational learning than memorisation of caches made by others. For example, the latter must be done from a distance and hence may require the ability to adopt an allocentric perspective, i.e. the ability to visualise the cache from the hoarder’s perspective.

**Significance statement:**

Members of the Paridae family are known to possess foraging techniques that are cognitively advanced. Previously, we have demonstrated that a non-hoarding parid species, the great tit *P. major*, is able to memorise positions of caches that they have observed marsh tits *P. palustris* make. However, it is unknown whether this cognitively advanced foraging strategy is unique to great tits or if it occurs also in other parids. Here, we demonstrated that “pilfering by observational memorization strategy” is not a general strategy in parids. We believe that such ability is important for a non-hoarding species such as the great tit and, most likely, birds owning many caches do not need this foraging strategy.

## Introduction

Many species in the passerine family Paridae (tits, titmice and chickadees) are well known to people in the northern hemisphere as they are common visitors to bird feeders and frequently nest close to humans. Relative to other small passerines, they are known to possess foraging techniques that are cognitively advanced; for example, some species have a large spatial memory capacity for cached food (e.g. Shettleworth 1983) and others the ability to use tools or a high ability to explore novel food sources such as sealed milk bottles (Fisher and Hinde 1949; Gosler and Clement 2007). This is logical if one considers that the species in this family possess relatively large brains compared to most other passerines (Lefebvre and Boogert 2010).

Most parids are non-migratory and spend the winter in boreal or temperate zones. Here, winter conditions can be harsh and, in cold winters, mortality will be high. As winter survival depends on effective foraging (Jansson et al. 1981), the selection for this will be strong. When it comes to wintering strategies, there is a clear dichotomy in the parid family. Most species are resident in year-round territories in which they rely on winter food that they have stored in autumn (Ekman 1989). In Europe, the exceptions to this strategy are the non-hoarding great tits *Parus major* and blue tits *Cyanistes caeruleus* (Ekman 1989). During the non-breeding season, these species are non-territorial forming large flocks that exploit all possible types of food sources that they may encounter.

Great tits are considered to be innovative foragers (Lefebvre and Boogert 2010; Cole et al. 2011; Cauchard et al. 2013; Brodin and Urhan 2015). For example, they have been recorded to use conifer needles to extract food from bark crevices (Gosler and Clement 2007). They are better observational learners and problem solvers than blue tits and marsh tits *Poecile palustris* (Sasvári 1979; Aplin et al. 2015), and they are surprisingly good at spatial memorisation (Brodin and Urhan 2014). The cognitive abilities of the hoarding species in the family, on the other hand, may be more focussed on massive memorisation of caching positions. Hoarding parids such as marsh tits and willow tits, *Poecile montanus*, stock their winter territories with large amounts of cached seeds and nuts that constitute an important part of their winter diet (Haftorn 1956; Jansson 1982). These species appear to be less explorative than the great tits, and they can even appear to be neophobic at novel food sources (Mönkkönen and Koivula 1993). One of us (AB) has much experience in feeding parids in forests. When a feeder is introduced in an area where bird feeding has not previously occurred, it may take months before local willow tits or marsh tits start visiting the feeder. If there are great tits around, however, these will typically investigate the feeder promptly and start eating from it almost immediately (AB, pers. obs.).

This dichotomy is apparent in the interaction between great tits and their relatives. Great tits are not food hoarders themselves but will follow their hoarding relatives in order to pilfer caches, a behaviour not observed in other parids (Gibb 1954; Gosler and Clement 2007). As they are substantially larger than other parids, they can easily displace hoarders directly after the food item has been stashed in its cache. Great tit cache pilfering, however, may be considerably more refined than this. Great tits can observe caching marsh tits from a distance, memorise cache positions and return later and steal the food (Brodin and Urhan 2014).

It is possible that this “observational memorisation of caches” is a unique foraging strategy of the great tit among parids. The American black-capped chickadee *Poecile atricapillus* is a close American relative to the marsh tit with a similar storing behaviour (Brodin 2005). In an experiment that was very similar to the ones in which Brodin and Urhan (2014, 2015) showed this ability in great tits, chickadees did not show any evidence of this (Baker et al. 1988). Neither did Hitchcock and Sherry (1995) detect such an effect in black-capped chickadees in a slightly different experiment.

On the other hand, all animals that have brains of the same size as the brains of passerine birds will be capable of some degree of observational learning. All passerines can probably learn foraging techniques, recognition of various food types and predators, etc. by observing conspecifics. Thus, it is possible that the observational memorisation ability that we have demonstrated in great tits (Brodin and Urhan 2014) may be a general ability in parids. The fact that Baker et al. (1988) did not detect it in black-capped chickadees could in that case depend on differences in experimental design or procedure.

Our aim with this study is to test if marsh tits are able to make the same type of memorisation of positions of caches made by other marsh tits as great tits. We will test this in the same laboratory set-up where we previously have tested great tits. Birds that have such ability should be more successful after the short interval (1 h) than after the long interval (24 h). After 24 h, they should still be more successful than in the control sessions.

## Methods

### Subjects

During the autumns of 2014 and 2015, we captured 17 marsh tits by using mist nets and playback near Höör (55° 56′ N, 13° 32′ E) in southern Sweden. We kept the birds under permit M213-11 from the Malmö-Lund regional ethical committee for animal experiments and housed them individually in 40 × 40 × 60 cm cages. We placed the cages on shelves on one side of the experimental room, with two cages on each shelf. Marsh tits occur pairwise in their territories, and we placed birds captured at the same location together so they could have visual and auditory contact with each other. The birds had ad libitum access to a crushed nut mix that contained sunflower seeds, peanuts and hemp nuts in their cage. In order to give the birds opportunity to handle living food, we provided them with mealworms daily. We also provided them with a suet cake that contained seeds. We added a commercial bird vitamin mixture in the birds’ drinking water and changed it daily. After the experiment, we released the birds at the same location where we had captured them.

### Experimental facility

We conducted the experiment in a 5 × 3 × 2.6 m indoor facility at the biology department at Lund University. The room had a computer-controlled light regime, which was set at a 10-h light/14-h dark cycle. The lights had a daylight spectrum and a 1-h dimming function, which allowed us to simulate dawn and dusk in the laboratory. The temperature in the room was held constant at 14 °C. The rationale for this was that the food hoarding intensity of marsh tits peaks in September/October when days may be 10 h long and 14 °C is a typical outdoor temperature around Lund and Höör.

See Brodin and Urhan (2014, Fig. 1) for a detailed figure of the laboratory. The entrance door to the laboratory led into a 2 × 1 m glass booth. We had equipped the glass of the booth with smoke-coloured foil, which made it possible to observe the birds without disturbing them. We had mounted the shelves for the bird cages on the wall adjacent to the observation booth. A wall separated the bird-housing compartment from the experimental arena. An observer in the booth had full view of both the experimental arena and the birds’ housing area. In front of each cage, there was a door that could be removed from its hinges to give the bird behind it full view of the arena from its cage. Next to each door, we had connected each cage to a small gate that led into the experimental arena through a short plastic tube. A slide shutter that we could open and close by remote control covered each gate. When a shutter was open, the bird in the cage behind it had free access to the arena. With this remote-controlled system, we did not have to handle the birds before or after the experimental sessions, a potentially very stressing experience for wild birds. To end a session, we turned off the lights in the experimental arena. The only light would then come from the gate to the bird’s home cage. The bird would then fly to the gate and leave the arena.

In the arena, we had positioned 10 boards (“artificial trees”) serving as caching substrates (see Fig. 1 in Brodin and Urhan 2014). The “trees” were 2.5 m tall and we had drilled 10 possible caching holes in each. Five centimetres below each caching hole, we had mounted a stick with a diameter of 8 mm that would serve as a perch for the birds. Above each hole, we had stapled a piece of cloth that we could pull up or down. When the cloth was fully down, it covered the caching hole completely. When it was up, the caching hole and its content were fully visible. We had positioned the trees opposite the bird housing area and the observation booth so that an observing bird would have full view of all possible caching sites regardless of its position in its cage. Obviously for the same reason, we had drilled the caching holes on the side of the trees that faced the observing bird. We had painted various figures and patterns in several colours on the artificial trees, walls, floor and doors of the birds’ housing area. The rationale was to create an environment that contains sufficient spatial information for the birds to memorise and make it possible for them to orientate themselves around the caching holes.

### Experimental procedure

Before we started the observational experiment, we trained six marsh tits to store and retrieve seeds in the hoarding arena. We trained 11 other marsh tits to search for food in the caching holes but did not allow these birds to store themselves. The rationale for this procedure was to minimise the risk that memories of their own caches would interfere with memorisation of observations when others cache. We used the birds that we had trained to both store and retrieve as demonstrators and the other 11 birds as observers. As we had a maximum of six birds in the cages at the same time, we would have different constellations of demonstrators and observers in the lab as the experiment progressed. Some of the observers took the demonstrator’s role after the trials that their roles as observers were completed. However, we did not use the demonstrator birds as observers since the memory of the positions of their own caches might have interfered with the memory of the caches they have observed. Two hours before the training and experimental sessions, we removed the food from both the observer’s and the demonstrator’s cage. We considered the training of demonstrators as completed when a bird readily would store five seeds and the training of observers as completed when a bird had learnt to search for food in the caching holes behind the cloths.

An experimental replicate consisted of two stages starting with the observation session. Before we started such a session, we placed a stool with a container of sunflower seeds in the centre of the arena. We then removed the door in front of the observer’s home cage to give it full view of the arena and the caching holes. As the slide shutter to the observer’s cages was shut, it could see but not enter the arena. Then, we opened the slide shutter to the demonstrator’s cage so that it could fly into the arena and start storing. Each observation session lasted until the demonstrator had stored five seeds or for a maximum of 15 min. In a few sessions, the demonstrator only stored four seeds. These are also included in the analyses, and this is the reason that we used proportions rather than numbers of seeds in our analyses. We stopped the caching session by turning off the lights in the experimental area. Directly after the demonstrator had returned to its cage, we closed the door in front of the observer’s cage so that it could no longer see the arena.

The second stage was the retrieval session. After either a 1- or a 24-h retention interval, we opened the slide shutter between the observer’s cage and the arena and allowed it to search for the demonstrator’s caches. We allowed it to search for 5 min and then turned the lights off so that the bird flew back into its cage. Before a retrieval session started, we randomly removed two out of five seeds to minimise the risk of satiation, which could affect the birds’ motivation to search for food. In order to make sure that the observers were motivated to start searching for the hidden caches, we deprived the birds of food before the retrieval sessions as we did before the caching sessions. The total time of food deprivation was 3 h, except for the early morning sessions that occurred after the 24-h retention intervals. The early morning 24-h retrieval sessions started right after the lights were turned on in the aviary. Since the birds do not feed overnight, the birds will be very hungry and motivated when the lights are turned on in the morning.

As a control, we allowed each observer to search for caches from a session it had not observed. Also, for the control sessions, we removed two out of five seeds to reduce the risk of satiation. We alternatively started with either control or experimental sessions for different birds. We also alternated between 1- and 24-h retention intervals as the first retrieval session. As in retrieval sessions, the birds were deprived of food for 3 h prior to control sessions. There was little need for blinded methods since it is very obvious to an observer whether a bird visits a caching hole or not. Not only has the bird to fly to the hole and perch there, it must also remove the piece of cloth that covers the hole to inspect it.

As in previous studies (Brodin and Urhan 2014, 2015), we used two measures to evaluate the birds’ performance. One was the proportion of correct retrieval attempts after the first 10 attempts, and the second was the number of erroneous attempts before the first correct one. Since a marsh tit could visit all 100 caching holes within 5 min, the performance during the whole retrieval session will not be a meaningful measure. We arcsine square root transformed all proportions, and unless otherwise stated, we used two-tailed paired *t* test to test for significance.

## Results

**Fig. 1:**
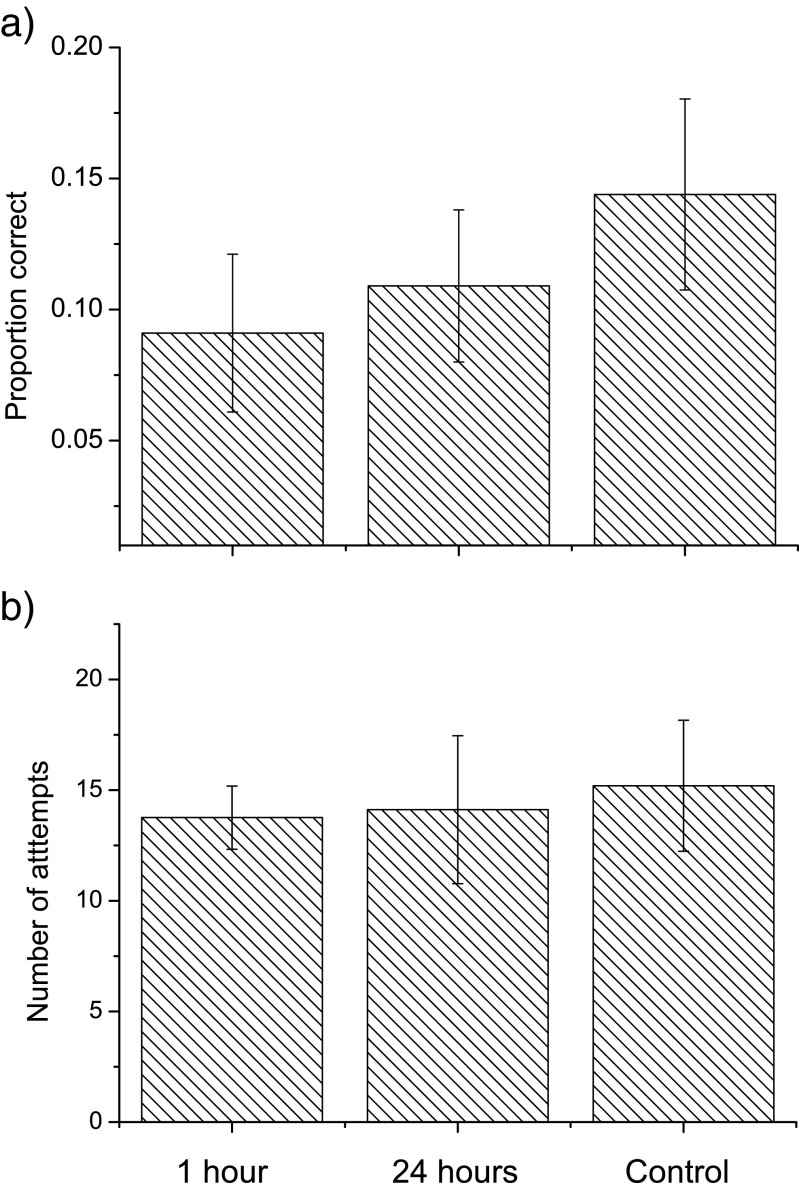
**a** Proportion of correct looks in the first 10 looks after 1-h, 24-h and control sessions. **b** Number of attempts before the first correct look in 1-h, 24-h and control sessions. The error bars are 95% confidence intervals

Neither the proportion of correct looks in the first 10 retrieval attempts after the 1-h retention interval (*t* = 1.275, df = 10, *p* = 0.231) nor the 24-h retention interval (*t* = 0.964, df = 10, *p* = 0.357) differed from the control sessions (Fig. 1a). Neither was the difference between the 1- and 24-h intervals significant (*t* = 0.245, df = 10, *p* = 0.811). It should be noted that even though we found no significant differences, the tendencies suggested by the means were in fact opposite to our predictions. The mean was highest in the control sessions and lowest after the 1-h retention interval (Fig. 1a).

Also with our alternative measure, the outcome tended to be opposite to our predictions as the birds’ mean performance was highest in the control sessions and lowest after the short 1-h interval (Fig. 1b). Neither of these differences were significant: 24 h vs control (*t* = 0.260, df = 10, *p* = 0.779), 1 h vs control (*t* = 0.584, df = 10, *p* = 0.571) and 24 vs 1 h (*t* = 0.113, df = 10, *p* = 0.911). This means that with both our measures, any tendencies were opposite to our predictions.

The mean number of retrieval attempts after the 1-h retention (47.2 ± SD 12.4) was significantly higher than in the control sessions (35.3 ± SD 8.03) (*t* = 3.375, df = 10, *p* = 0.007). It tended to be higher after 1 h than after 24 h (34.2 ± SD 17.4), but this difference was not significant (*t* = 1.869, df = 10, *p* = 0.091). However, as it could be argued that memory only can decay (and not improve) over time, a one-tailed test could be employed here and then this difference would be significant. There was no difference between the mean number of retrieval attempts after 24 h and in the control sessions (*t* = 0.199, df = 10, *p* = 0.845).

If we assume that the effect is as strong in marsh tits as it was in great tits (Brodin and Urhan 2014), we would have had a power of 0.99 for the test between the 1-h interval sessions and the control sessions and 0.75 for the 24-h sessions vs the control sessions (calculated on the data in Brodin and Urhan 2015). As the tendencies in Fig. 1 were opposite our predictions, we would get negative effect sizes and refrain from making power analyses of our tests.

## Discussion

In contrast to great tits (Brodin and Urhan 2014, 2015), marsh tits failed to memorise the positions of caches that they had seen others make in the very same laboratory set-up. This result was consistent in both our measures “proportion of correct attempts in the first 10 looks” and “the number of looks before the first correct attempt”. To us, this indicates that marsh tits do not use this type of observational memorisation as a foraging strategy in the same way as great tits. In retrospect, this is logical. Why should a hoarder that easily can memorise hundreds of its own caches invest in the difficult task of memorising caches that others have made? For a non-hoarder such as the great tit, on the other hand, a few memorised marsh tit caches could be crucial for survival.

The lack of such a capacity may still seem odd as marsh tits are specialised hoarders, probably capable of memorising hundreds or even thousands of their own caches (Haftorn 1959; Cowie et al. 1981). The relative volume of the hippocampal complex is larger in marsh tits than in great tits (Krebs et al. 1996; Lucas et al. 2004), and an enlarged hippocampus is usually associated with advanced spatial cognition (Gaulin et al. 1990; Sherry et al. 1993; Gaulin 1995). Therefore, one may expect marsh tits to be more successful than great tits in various types of spatial memory tasks. However, the memorisation of other’s caches from some distance probably involves different cognitive processes than the “standardised” routine memorisation of their own caches that hoarding parids make (Watanabe and Clayton 2007).

The characteristic behaviour of a food-storing parid usually starts with the inspection of some potential caching locations. As the hoarder approaches a suitable location, it will have opportunity to observe the site from various angles and distances. After making a cache, the bird performs a close visual inspection of the cache with one eye by turning its head 90° (Gibb 1954; Sherry et al. 1981). This process allows the hoarder to memorise the changing visual perspectives when approaching the location as well as the close-up characteristics of the cache and the nearby landmarks. To memorise a cache position that another individual has made, on the other hand, will be a different process (Watanabe and Clayton 2007).

A great tit that observes a caching bird with the intention of memorising the cache position must be perched sufficiently far away so as not to alert the hoarder. This means that the close inspection of a cache that a hoarder makes will not be possible and that the observer must rely on memorisation from an allocentric perspective (Brodin and Urhan 2014). Additionally, an observer has no control over the selection of the location and it cannot approach the cache in the way a hoarder does. This distance memorisation is probably a difficult task as it previously has been demonstrated only in corvids (e.g. Heinrich and Pepper 1998; Watanabe and Clayton 2007), and these are supposed to be the cognitive “giants” among birds (e.g. Emery and Clayton 2004; Kabadayi et al. 2016).

It might be argued that there could have been an effect but our sample size (11 observers) was too small to detect it. We see this as very unlikely, however, as the tendencies of the means went opposite to our predictions (Fig. 1). Since memory must decay over time, it would be logical to assume that the birds should have performed better in the 1-h sessions compared to the 24-h sessions and, of course, that they should have performed better in both these sessions than in the control sessions. In the very same laboratory set-up, we had no difficulty in detecting such an effect previously in 16 great tits (Brodin and Urhan 2014). In the great tit study, the effect was strong giving a power over 0.9 for both the 1- and 24-h intervals.

This also suggests that if there was an effect also in marsh tits, it would be smaller than in great tits. As the tendencies of our 11 marsh tits were opposite to any expected effects, we see it as rather meaningless to increase the sample size by testing more birds. Even if we assume that the effect in marsh tits is as strong as in great tits, we would need to add 100 to 150 birds to our present sample to get a significant effect. Therefore, it seems meaningless and time consuming to test more individuals in order to search for an effect that still could not change the outcome of the study with reasonable sample sizes.

Why then do we think that marsh tits do not have this type of observational memorisation ability? As marsh tits are skilled food hoarders themselves, they can probably increase fitness more easily by additional storing of their own caches than by time-consuming and difficult “spying” on other hoarders. It is even possible that such memorisation would interfere with memories of their own caches (Crystal and Shettleworth 1994; Kamil and Gould 2008).

It is hence possible that species with many of their own caches do not need this ability. To our knowledge, there are no comparative studies of caching memory vs observational memory in parids but, in corvids, less specialised hoarders seem to have better observational memorisation capacity than more specialised hoarders. Mexican jays *Aphelocoma wollweberi*, a less specialised hoarder, was less accurate when retrieving their own caches than Clark’s nutcrackers *Nucifraga columbiana*, a highly specialised hoarder (Vander Wall and Balda 1981; Bednekoff and Balda 1996). When it came to observational memorisation of caches made by other individuals, however, they were more accurate than the nutcrackers (Bednekoff and Balda 1996).

Here, by no means do we claim that marsh tits are not capable of observational learning. What we have demonstrated here is that marsh tits did not memorise cache locations that they have observed others make. Neither do we claim that marsh tits do not pilfer caches made by other individuals, only that they do not use the same memorisation technique as great tits when they do this. Cache pilfering by conspecifics may be high in marsh tits (Cowie et al. 1981; Sherry et al. 1982). Such high levels of pilfering will occur because birds such as marsh tits will search systematically for cached items around rich food sources such as bird feeders (Brodin 1992).

There are several factors that could have affected the marsh tits’ performance negatively. The birds were kept and tested under artificial laboratory conditions which may have decreased their performance. This is unlikely, however, since great tits performed well in the same set-up and other marsh tits have performed well in previous experiments in this set-up (see below). Another possibility is that the marsh tits were not sufficiently motivated when they observed their conspecifics hoarding. This also seems less likely to us as the procedure with food deprivation before the experimental sessions has worked well to motivate marsh tits before (Brodin and Urhan 2013). We think that the fact that the observer marsh tits made more retrieval attempts after the 1-h retention interval compared to both control and 24-h sessions shows that the birds were motivated. We interpret this difference as that the observers remembered that other individual marsh tits recently (1 h ago) had been storing in the arena and that the probability that there will be food in the caching sites then must be high.

Also, we have tested marsh tits in various food-hoarding experiments before in the same laboratory (Brodin and Urhan 2013; Urhan and Brodin 2015) and, during these experiments, marsh tits were highly motivated to both store and retrieve food. Moreover, they were able to memorise positions of their own caches. Also, our subjective impression is that the observer marsh tits always seemed to be responsive and interested in the behaviour of the demonstrator marsh tit. We did not collect data on this, but the typical behaviour of an observing marsh tit when we released the demonstrator into the hoarding arena was that it perched still and appeared to lock its gaze on the demonstrator bird. Previous studies have demonstrated that parids typically tend to observe each other’s foraging activity and modify their own accordingly (Krebs et al. 1972).

In conclusion, marsh tits did not seem to be able to memorise the positions of caches made by conspecifics. This result agrees with those of similar previous studies on a food-hoarding parid, the black-capped chickadee (Baker et al. 1988; Hitchcock and Sherry 1995). This makes it likely that the special observational memorisation ability of great tits is not a general ability of all parid species.
